# Seroprevalence of three paramyxoviruses; Hendra virus, Tioman virus, Cedar virus and a rhabdovirus, Australian bat lyssavirus, in a range expanding fruit bat, the Grey-headed flying fox (*Pteropus poliocephalus*)

**DOI:** 10.1371/journal.pone.0232339

**Published:** 2020-05-06

**Authors:** Wayne S. J. Boardman, Michelle L. Baker, Victoria Boyd, Gary Crameri, Grantley R. Peck, Terry Reardon, Ian G. Smith, Charles G. B. Caraguel, Thomas A. A. Prowse

**Affiliations:** 1 School of Animal and Veterinary Sciences, University of Adelaide, Adelaide, South Australia, Australia; 2 CSIRO Health and Biosecurity Business Unit, Australia Animal Health Laboratory, Geelong, Victoria, Australia; 3 CSIRO, Australian Animal Health Laboratory, Geelong, Victoria, Australia; 4 South Australia Museum, Adelaide, South Australia, Australia; 5 Zoos South Australia, Adelaide, South Australia, Australia; 6 School of Mathematical Sciences, University of Adelaide, Adelaide, South Australia, Australia; University of Pretoria, SOUTH AFRICA

## Abstract

Habitat-mediated global change is driving shifts in species’ distributions which can alter the spatial risks associated with emerging zoonotic pathogens. Many emerging infectious pathogens are transmitted by highly mobile species, including bats, which can act as spill-over hosts for pathogenic viruses. Over three years, we investigated the seroepidemiology of paramyxoviruses and Australian bat lyssavirus in a range-expanding fruit bat, the Grey-headed flying fox (*Pteropus poliocephalus*), in a new camp in Adelaide, South Australia. Over six, biannual, sampling sessions, we quantified median florescent intensity (MFI) antibody levels for four viruses for a total of 297 individual bats using a multiplex Luminex binding assay. Where appropriate, florescence thresholds were determined using finite mixture modelling to classify bats’ serological status. Overall, apparent seroprevalence of antibodies directed at Hendra, Cedar and Tioman virus antigens was 43.2%, 26.6% and 95.7%, respectively. We used hurdle models to explore correlates of seropositivity and antibody levels when seropositive. Increased body condition was significantly associated with Hendra seropositivity (Odds ratio = 3.67; p = 0.002) and Hendra virus levels were significantly higher in pregnant females (p = 0.002). While most bats were seropositive for Tioman virus, antibody levels for this virus were significantly higher in adults (p < 0.001). Unexpectedly, all sera were negative for Australian bat lyssavirus. Temporal variation in antibody levels suggests that antibodies to Hendra virus and Tioman virus may wax and wane on a seasonal basis. These findings suggest a common exposure to Hendra virus and other paramyxoviruses in this flying fox camp in South Australia.

## Introduction

The emergence of zoonoses from wildlife represents an increasingly significant threat to global public health [1]. Bats (Order Chiroptera) are the reservoir host of several significant groups of emerging zoonotic viruses including the paramyxoviruses, (e.g. Hendra virus and Nipah virus), coronaviruses, filoviruses and lyssaviruses [2–5]. In Australia, spill-over of three viruses associated with bats of the genus *Pteropus*, also known as flying foxes, has led to morbidity and mortality in domestic animals and humans. They include two paramyxoviruses, Hendra virus and Menangle virus, and a rhabdovirus, Australian bat lyssavirus [6–10]. Research into the ecology of these viruses led subsequently to the discovery of several new paramyxoviruses, including Cedar virus, Hervey virus, Yeppoon virus, Grove virus, Teviot virus [11] and Tioman virus [12]. Tioman virus, closely related to Menangle virus [8] is the only one of these viruses to be associated with disease. It has been associated with sub-clinical infection in humans and still births and fetal abnormalities in pigs [13].

The Grey-headed flying fox (*Pteropus poliocephalus*), one of four species of flying foxes found on mainland Australia, is classified nationally as vulnerable under the *Environment Protection and Biodiversity Conservation Act 1999* [14]. The geographical distribution and migration of Grey-headed flying foxes and other pteropodids is dictated by the distribution and phenology of food plants. These bats regularly move long distances in search of ephemeral floral and fruit resources in native forests [15,16]. Aggregations of flying foxes can increase rapidly during highly productive flowering events [17]. Recently, Grey-headed flying foxes were distributed from Ingham in Queensland along the coastal belt of eastern Australia to Melbourne in Victoria. As natural food resources have declined coincident with substantial (*c*. 75%) loss of native forest throughout the south-eastern coastal areas of Australia, Grey-headed flying foxes have sought alternative food sources, sometimes forming new colonies in urban landscapes [18,19].

Habitat loss and fragmentation reduce not only the quantity of food available to wildlife, but also the connectivity of foraging patches, particularly if seasonally important resources have been removed [16]. In contrast, anthropogenic resource subsidies, which favour monoculture (e.g., fruit orchards) and introduced species, change the composition and seasonality of available food and the overall nutritional landscape [16]. Recently, Grey-headed flying foxes formed camps in Canberra and western parts of Victoria and, during the 2010 winter, approximately 1300 individuals migrated to Adelaide, South Australia, thereby expanding the former range of the species. Since that time, the population in Adelaide’s Botanic Park, which is a popular recreational location, has increased to approximately 20,000 individuals due to births and continued immigration(November 2019), despite seasonal emigration and substantial bat mortality events during extreme heat waves in summer. Concerns have been raised that the bat camp may constitute a biohazard to the public and to domestic animals. Indeed, Australian bat lyssavirus was detected in a Grey-headed flying fox from the camp in 2012 [20]. Since then, another twenty-six Grey-headed flying foxes from the camp were opportunistically tested for the virus of which none tested positive. However, uncertainty remains about the endemicity of Australian bat lyssavirus in the Adelaide camp.

When investigating the infection dynamics of emerging viruses in bat colonies, direct viral detection and identification is important but is technically limited due to restricted distribution of the virus in organs and transient viral shedding in biological fluids. Complementing virus detection, the exposure to specific viruses can be measured by detecting antibodies against those viruses in bat sera. Antibodies are generally present for months or even years even if the virus is scarely distributed or even after it is cleared from the animal. As a result, viral seroprevalence monitoring has often been the first line of investigation for emerging bat zoonoses [21–25]. However, interpreting serological results is challenging [26] in part due to variation in the magnitude and longevity of antibody responses to different viruses, and the time of collection of serum post infection [27]. Furthermore, antibodies may cross-react with or cross-neutralize related viral antigens, which can limit the specificity of assays.

Serum viral neutralization tests (SNTs) have been considered the reference method for detecting specific antibodies to Hendra virus [28]. However, the use of SNTs is logistically constraining because the highest level of biocontainment (Biosafety level 4) is required to maintain the live viral cultures used for the neutralization assays. Instead, IgG enzyme-linked immunosorbant assays (ELISAs) and Luminex based assays [29] have been favoured because they can be performed under standard biosafety conditions [30]. Luminex based fluorescent microsphere binding assays [29] are a sensitive method for detection and quantification of antibodies against Hendra and Nipah viruses [15, 22, 31, 32] and Australian bat lyssavirus [33] in bat sera. The target antigen for Hendra virus and Cedar virus is recombinant soluble G protein [32] while the target antigens for Tioman virus and Australian bat lyssavirus are nucleoproteins. Luminex assays have been used internationally to detect henipavirus antibodies in bats and other species; including West African fruit bats and domestic pigs [22,34–36], pteropodid bats in Papua New Guinea [15] and *Pteropus vampyrus* bats in Indonesia [37].

Serological evidence of infection with Hendra virus has been shown in all four species of pteropodid bat that occur on mainland Australia, throughout their respective ranges [21, 38]. There is evidence to suggest that two species, namely the Black flying fox (*Pteropus alecto*) and the Spectacled flying fox (*P*. *conspicillatus*), play the most active role in the transmission of Hendra virus to horses [38]. Hendra virus is shed in the urine, an important vehicle for transmission in Black flying foxes [10, 38–39], and the virus has been detected in Grey-headed flying fox uterine fluid which provides evidence for possible transmission at birthing period which lasts from late September to early December [40] in this species [41, 42].

Here, we surveyed the exposure of Adelaide’s recently established Grey-headed flying fox population to protein antigens of Hendra virus, Cedar virus, Tioman virus and Australian bat lyssavirus over a three year period. We used results from Luminex antibody binding assays to develop a finite-mixture model to identify thresholds for defining seropositive flying foxes to characterise seroprevalence for these four viruses. Next, we used a negative-binomial hurdle model and investigated individual-level correlates of (i) seropositivity and (ii) antibody level following seroconversion. We hypothesised that Hendra virus seroprevalence and antibody levels would be associated with reproductive status as previously reported [21,23] and that Australian bat lyssavirus seroprevalence would be apparent given the prior finding of an individual carrying the virus in 2012 [20].

## Materials and methods

We followed the Consortium for the Standardization of Influenza Seroepidemiology (CONSISE) guidelines [43] for the reporting of seroepidemiologic studies. Animal Ethics approval was obtained from The University of Adelaide (S-2015-028) and a wildlife scientific permit from the SA Department of Environment and Water (M-23671-1,2 and 3) prior to commencement of this project.

### Sampling

#### Study population

**Fig 1:**
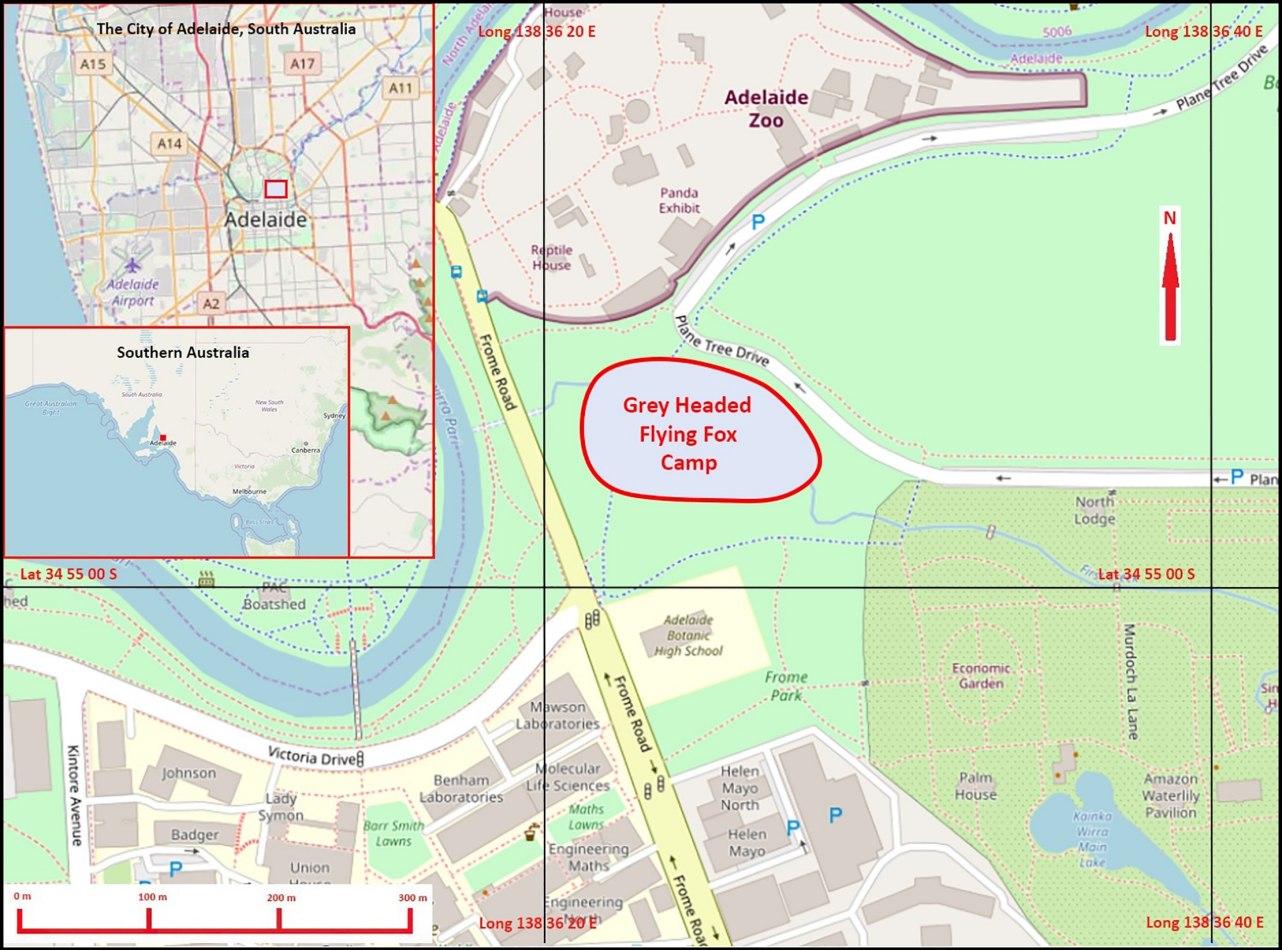
Location of the Grey-headed flying fox camp in Adelaide’s Botanic Park and relationship to central Adelaide and Southern Australia.

The target and source population were the grey-headed flying foxes from the only known camp in SA (Fig 1) and established in Adelaide’s Botanic Park [approximate GPS coordinates: 34°54'56 S, 138°36'24 E]. The camp was sampled over six surveys at approximately six-month intervals between August 2015 (winter) and February 2018 (summer), with the aim of trapping > 50 animals per survey, which constituted our study population.

Location and extent (red line) of the Grey-headed flying fox camp in Adelaide’s Botanic park, showing proximity to Adelaide Zoo where bats were processed. Insets illustrate central Adelaide and Southern Australia to show geographical relationships. Geodata from OpenStreetMap was downloaded via the Maperitive application and the map was rendered with further information supplied by the author.

#### Bat handling, serum and data collection

Study animals were captured at the roost site using 12 or 18 m long mist nets (Ecotone, Gdynia, Poland) installed beneath the camp. Mist nets were raised 20 m above the ground before bats returned from their nightly foraging activity. As each bat became entrapped, the net was lowered, the bat handled with care using thick leather gloves to assure handlers’ health and safety, then transferred into pillow cases and relocated to the Animal Health Department of the adjacent Adelaide Zoo. The net was then elevated to 20 m above the ground to catch further bats. This continued until all bats had returned to the camp. Isoflurane (Isoflurane, Laser Animal Health) was used to anaesthetise bats during data and sample collection following the protocol described by Jonsson et al, 2004 [44]. Each bat was permanently identified using a passive integrated transponder tag (Trovan, Microchips Australia Pty, Keysborough, Victoria) inserted subcutaneously between the scapulae. A small amount of fur was clipped from the chest to rapidly identify recaptures at a given survey. In order to prevent dehydration during their short term confinement, 20–40 mL Hartmann’s fluid were injected subcutaneously between the scapulae. Approximately 3–4 mL of blood was collected via venepuncture of the propatagial or brachial vein into 4 mL serum tubes using 22-gauge needles and 3–5 mL syringes. These were allowed to clot overnight at room temperature and then at 4°C before centrifugation (5,000 rpm for 5 minutes) and separation of serum, which was subsequently stored at -80°C. After sampling, bats were placed into pillowcases to fully recover from the anesthesia before release into the camp.

For each bat, we recorded: (i) sex, (ii) body weight (BW; g), (iii) body condition score (scale of 1 to 5 based on physical palpation of the pectoral musculature by the same person), (iv) forearm length (FAL; mm); elbow to wrist length using vernier callipers, (v) estimated age as described by Hall and Richards [40] (including teeth wear, nipple size for females and enlarged penis/testes for males), (vi) reproductive status (for females; pregnant vs not pregnant by abdominal palpation, lactating vs non lactating by expression of milk and for males; enlarged penis/testes vs small penis/testes). For an objective estimate of the body condition, we also derived a body condition index (BCI) for each individual, calculated subsequently as BCI = 1,000*(BW/FAL^2^).

### Serology for Hendra virus, Cedar virus, Tioman virus and Rabies virus

Serum samples experienced two freeze/thaw cycles prior to testing. Antibodies against Hendra virus, Cedar virus, Tioman virus, and Australian bat lyssavirus antigens were detected at the Australian Animal Health Laboratory in Geelong, Victoria using multiplex microsphere assays (Luminex, Austin, USA) as described previously [29]. The conformational status of the viruses used were the following; soluble native-like oligomeric G envelope glycoproteins of HeV and CedV (sG_tet_) were produced from stable expressing FreeStyle^TM^ 293F cell lines [45,46], Tioman virus was a nucleocapsid protein expressed in the yeast *Saccharomyces cerevisiae* [47], and Australian bat lyssavirus was a nucleocapsid protein prepared in E.coli [48]. Briefly, prior to analysis, serum samples were first heat treated at 56°C for 30 minutes to inactivate complement then the assay proteins were coupled to individual microsphere bead sets, of predetermined numbers of magnetic beads, MagPlex^®^ (Luminex, Northbrook, USA). These were added to each well and then mixed with bat sera at a dilution of 1:50. The bound antibody was detected using biotinylated Protein A (Pierce, Rockford, USA) together with biotinylated Protein G (Pierce, Rockford, USA) followed by streptavidin–phycoerythrin (Qiagen Pty Ltd, Australia). The assay was read using a Bio-Plex Protein 200 Array System integrated with Bio-Plex Manager Software (v 6.2) (Bio Rad Laboratories, CA, USA) calibrated on the high setting. Each sample was tested in a well with thousands of beads and the florescence results of 100 beads were recorded as the median florescent intensity (MFI) that excludes outliers and are correlated with antibody concentration. Positivity thresholds for the Luminex serological assay have not been defined for Australian flying foxes due to the lack of negative and single-infection control serum [32] and were therefore estimated using finite mixture modelling (see below).

### Statistical analysis

#### Demographic analysis

Two-sample t-test statistics were used to identify any differences in BW, FAL and BCI across demographic classes (sub-adult males and females and adult males and adult pregnant and non pregnant females) and between winter and summer.

#### Estimating MFI thresholds for classifying seropositive animals

MFI values were log-transformed prior to analysis to approximate a normal distribution and enable parametric analyses. We used finite mixture modelling in the statistical package Stata v15.1 (College Station, Texas, USA) to identify the presence of more than one sub-population under the assumption of normal distribution. Models assuming up to three mixed distributions were run and their parsimony compared using Akaike Information Criterion (AIC) and Bayesian Information Criterion (BIC). The model with the lowest AIC and/or BIC was selected as final. When the single distribution model fitted best, the distribution was assumed to be the non-seroconverted bats if MFI values were in the lower end of the range and seroconverted bats if in the higher end of the range. When the two distributions model fitted best, the distributions were considered as the non-seroconverted and the seroconverted bats, respectively, according to their values’ range. When the three distributions model fitted best, the distribution with the lowest value range was considered as the non-seroconverted bats and the other two distributions (with higher value ranges) as two sub-groups of seroconverted bats. When two distributions fully overlapped, these were considered as one single distribution because the readings had no discriminative ability. For best fit models with more than one mixed distribution that overlapped partially, threshold values were determined visually at the MFI value for which two predicted normal distributions intersected. These threshold values were used to classify (imperfectly) individual bats as ‘seronegative’ or ‘seropositive’ (or ‘intermediate positive’ when three distributions were identified).

#### Hurdle modelling of seropositivity and antibody levels

To investigate correlates of seropositivity and MFI levels, we used a hurdle regression model which included two components; (i) the ‘hurdle’ component, which modelled the probability of being seropositive (as defined using the estimated lower threshold value); and (ii) a negative binomial count component, which modelled the antibody value (expressed as MFI) conditional on seropositivity. Explanatory variables investigated were those measured or observed during the trapping i.e. sex (male or female), age class (subadult < 2.5 years or adult ≥ 2.5 years), body weight (g), forearm length (mm), catching session (1–6), season (winter or summer), pregnancy status, lactation status, and body condition index (BCI). However, we excluded the effect of season from the final model due to strong collinearity between season and time of survey. Hurdle models were implemented within the *R* software for statistical computing (version 3.2.3) using the package *pscl*. The extent of co-seropositivity for all four viruses was also assessed using the negative binomial hurdle regression model investigating the same explanatory variables.

#### Demonstration of zero seroprevalence for Australian bat lyssavirus

The probability that the Adelaide camp is free from Australian bat lyssavirus was estimated using the historical survey analysis outlined by Cameron (2014) [49]. The probability of freedom from Australian bat lyssavirus was uncertain before the first survey and a prior value of 50% was used. In the absence of published information on the diagnostic accuracy of the multiplex Luminex assay, we optimistically assumed that this method had perfect accuracy for Australian bat lyssavirus antibodies. Similarly, little information is available on differential risks of Australian bat lyssavirus exposure across bat demographics. Therefore, the risk of Australian bat lyssavirus **‘**exposure’ was assumed constant across the bat camp strata (i.e. risk independent modelling). The ‘open’ nature of our study population was taken into account by including a ‘between-survey’ probability of exposure from and/or introduction of immigrating exposed bats into the model. Freedom from Australian bat lyssavirus seroprevalence was deemed achieved if the estimated probability of freedom was ≥ 95%.

## Results

### Demographic information

A total of 301 Grey-headed flying foxes were captured over six surveys. Four individual flying foxes were recaptured once each during this period. Demographics including sex, age, weight, forearm length and BCI varied across seasons reflecting the seasonality of the species’ reproduction and feeding opportunities (Tables 1 and 2). Approximately, two thirds were females and two thirds were adults. The overall percentage of adults was similar between sexes (61.7% vs 69.9%). Among the adult females, 31.9% (37/116) were pregnant at capture. Sex and age-class representation was similar across sampling and seasons. Overall the BCI was higher in winter (mean = 28.2, range = 20.2–36.1) than summer (mean = 25.3, range = 13.8–35.8) (+ 2.9 units, p < 0.001) mainly driven by the sub-adult BCI being higher in winter (n = 52, mean = 25.8, range = 20.2–29.6) than summer (n = 52, mean = 21.1, range = 13.8–29.2) (+4.7 units, p < 0.001).

**Table 1:** Summary demographic statistics–all captures.

	BW (g)			FAL (mm)			BCI		
Demographic Classes	n	Mean (SD)	Range	n	Mean (SD)	Range	n	Mean (SD)	Range
Sub-adult (all)	106	550 (115.4)	266–772	104	153.0 (7.3)	127.0–171.0	104	23.5 (3.6)	13.8–29.6
Sub-adult Females	72	566 (110.8)	291–739	70	153.8 (6.8)	140.0–170.5	70	23.9 (3.6)	13.8–29.6
Sub-adult Males	34	517 (119.5)	266–772	34	151.7 (7.8)	127.3–162.6	34	22.6 (3.4)	16.4–29.2
Adult (all)	195	764 (90.0)	563–1,008	193	164.0 (4.8)	152.0–176.8	104	23.5 (3.6)	13.8–29.6
Adult Females	116	743 (78.7)	563–1,005	114	163.0 (4.7)	152.0–174.4	114	27.9 (2.8)	22.0–35.1
Pregnant	37	792 (91.6)	600–1005	37	162.3(5.0)	152.0–171.4	37	30.0 (2.7)	22.8–34.9
Not Pregnant	79	720 (60.0)	563–963	79	164.0 (4.6)	154.0–173.0	79	27.0 (2.3)	22.0–35.2
Adult Males	79	794 (96.0)	585–1,008	79	165.2 (4.7)	155.1–176.8	34	29.1 (2.8)	22.6–36.1
All Females	188	676 (126.1)	291–1,005	184	159.5 (7.2)	140.0–173.4	184	26.4(3.7)	13.8–35.2
All Males	113	711 (164.2)	266–1,008	113	160.6 (9.0)	127.3–176.8	113	27.1 (4.2)	16.4–36.1
Total	301	689 (142.4)	266–1,008	297	160.0 (8.0)	127.3–176.8	297	26.7 (3.9)	13.8–36.1

Summary demography statistics (number (n), mean, standard deviation (SD), range) of 301 Grey-headed flying foxes captured over six surveys between September 2015 and February 2018 from the Adelaide Camp, Botanic Park, Adelaide. BW = Body weight, FAL = forearm length, BCI = body condition index = 1,000*(BW/FAL^2^)

**Table 2:** Summary demographic statistics–winter versus summer.

	WINTER									SUMMER								
	BW (g)			FAL (mm)			BCI			BW (g)			FAL (mm)			BCI		
Demographic Classes	n	Mean (SD)	Range	n	Mean (SD)	Range	n	Mean (SD)	Range	n	Mean (SD)	Range	n	Mean (SD)	Range	n	Mean (SD)	Range
Sub-adult	53	628.6 (42.8)	516–695	52	156.3 (4.1)	146.0–166.0	52	25.8 (2.0)	20.2–29.6	53	472(112.0)	266–772	52	149 (7.8)	127.0–161.0	52	21.1(3.3)	13.8–29.2
Females	40	629 (44.5)	516–695	39	156.2 (4.2)	147.0–166.0	39	25.9 (2.1)	20.2–29.6	32	486 (118.0)	291–739	31	150.5 (8.0)	140.0–170.5	31	21.3 (3.5)	13.8–29.0
Males	13	626 (38.6)	559–675	13	156.4 (3.8)	147.8–162.0	13	25.6 (1.6)	22.7–28.4	21	449 (100.7)	266–772	21	146.1 (7.0)	127.3–162.6	21	20.8 (2.8)	16.4–29.2
Adult	90	790 (99.2)	585–1008	90	163.3 (5.0)	152.0–173.0	90	29.5 (2.9)	22.6–36.1	105	741 (77.3)	563–992	103	164.5 (4.6)	153.5–176.8	103	27.4 (2.4)	22.0–35.8
Females	46	780 (92.5)	600–1005	46	162.1 (4.9)	152.0–171.4	46	29.6 (2.8)	22.8–34.9	70	719 (56.7)	563–963	68	163.8 (4.5)	153.5–173.4	68	26.8 (2.2)	22.0–35.2
Pregnant	37	792 (91.6)	600–1005	37	162.3 (5.0)	152.0–171.4	37	30 (2.7)	22.8–34.9	N/A	N/A	N/A.	N/A.	N/A.	N/A.	N/A.	N/A.	N/A.
Not Pregnant	9	732 (84.9)	645–844	9	161.1 (4.4)	155.1–168.4	9	28.2 (2.8)	24.1–32.6	70	719 (56.7)	563–963	68	164 (4.5)	154.0–173.0	68	26.8 (2.2)	22.0–35.2
Males	44	800 (105.8)	585–1,008	44	164.6 (4.8)	155.1–173.1	44	29.4 (3.0)	22.6–36.1	35	787 (82.9)	622–992	35	165.8 (4.6)	158.1–176.8	35	28.6 (3.1)	24.0–35.8
Total Females	86	710(105.5)	516–1,005	85	159.4 (5.4)	147.0–171.4	85	27.9 (3.1)	20.2–34.9	102	646 (134.7)	291–963	99	160 (8.5)	140.0–173.0	99	25.1 (3.7)	13.7–35.1
Total Males	57	761 (119.9)	559–1,008	57	162.8 (5.7)	147.8–173.1	57	28.6 (3.2)	22.6–36.1	56	660 (187.4)	266–992	56	158.5 (11.1)	127.3–176.8	56	25.7 (4.6)	16.4–35.8
TOTAL	143	730 (113.8)	516–1,008	142	160.7 (5.8)	147.0–173.1	142	28.2 (3.1)	20.2–36.1	158	651 (155.0)	266–992	155	159.2 (9.5)	127.3–176.8	155	25.3 (4.0)	13.8–35.8

Seasonal demography statistics (number (n), mean, standard deviation (SD), range, winter and summer) of 301 Grey-headed flying foxes captured over six surveys between September 2015 and February 2018 from the Adelaide Camp, Botanic Park, Adelaide. BW = Body weight, FAL = forearm length, BCI = body condition index = 1,000*(BW/FAL)^2^

### Serology thresholds and serological prevalence

**Fig 2:**
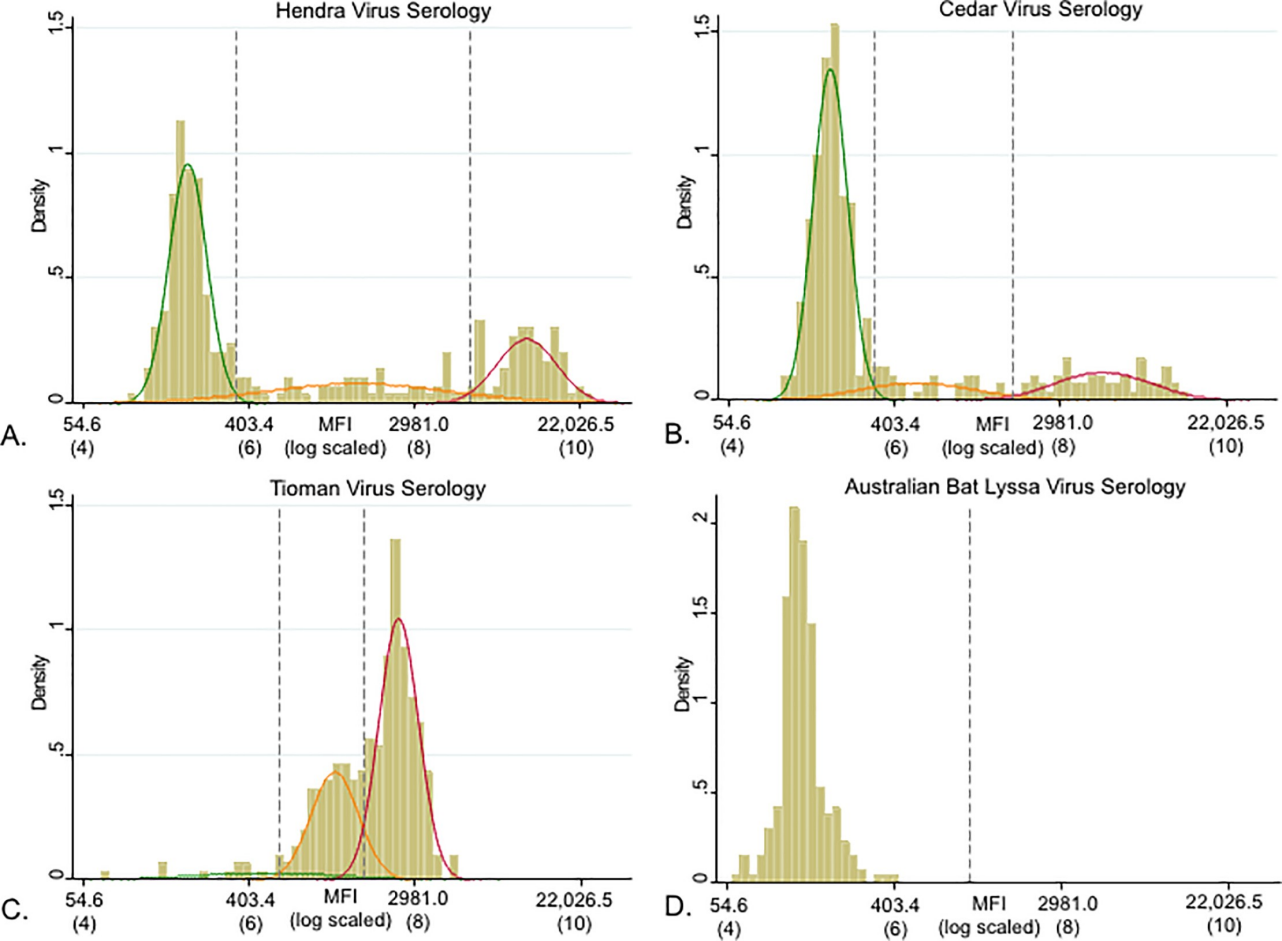
Density histogram and overlaid mixture of modelled distributions. Density histogram and overlaid mixture of modelled distributions for MFI and the natural log MFI of A) Hendra virus, B) Cedar virus, C) Tioman virus and D) Australian bat lyssavirus serological readings. Thresholds (dashed vertical lines) correspond to the intersection between a pair of predicted distributions. Upper and lower thresholds for the Hendra virus serology were determined as the natural antilogorithm of 5.85 and 8.67, respectively (MFI 347 and 5,825, respectively). Upper and lower thresholds for the Cedar virus serology was determined as the natural antilogorithm of 5.76 and 7.44 respectively (MFI 317 and 1,702 respectively). Upper and lower thresholds for the Tioman virus serology was determined as the natural antilogorithm of 6.37 and 7.38 respectively (MFI 584 and 1,603 respectively). Upper and lower thresholds could not be calculated for Australian bat lyssavirus as it was determined that all animals belong to the same exposure sub-population.

Multiplex serology was conducted on 301 serum samples (comprising 297 individual bats, with 4 recaptures). With the exception of Australian bat lyssavirus, these assays yielded multi-modal distibutions for log-transformed MFI (Fig 2). Three mixed distributions were identified for Hendra virus, Cedar virus and Tioman virus, and two cut-offs, a lower and upper, were determined visually (Fig 2). Upper and lower thresholds for the Hendra virus serology were determined as the natural antilogorithms of 5.85 and 8.67, respectively (MFI 347 and 5825, respectively). Upper and lower thresholds for the Cedar virus serology were determined as the natural antilogorithms of 5.76 and 7.44, respectively (MFI 317 and 1702, respectively). Upper and lower thresholds for the Tioman virus serology were determined as the natural antilogorithms of 6.37 and 7.38 respectively (MFI 584 and 1603, respectively). A single distribution of assumed non-seroconverted animals was identified for Australian bat lyssavirus. Using the lower threshold values, 26.6% of the bats were seropositive for Cedar virus, 43.2% of the bats for Hendra virus and 95.7% of the bats for Tioman virus were seropositive (Table 3).

**Table 3:** Lower threshold MFI scores with corresponding % seroprevalence.

Virus	Lower MFI threshold (log MFI)	% seroprevalence (Binomial exact 95% CI)	MFI median values (range) for seropositive animals
Hendra virus	347 (5.85)	43.2% (37.5%-49%)	6,813 (353–23,922)
Cedar virus	317 (5.76)	26.6% (21.7%-31.9%)	3,074 (326–13,759)
Tioman virus	584 (6.37)	95.7% (92.7%-97.7%)	2,121 (629–4,972)
Australian bat lyssavirus	Na	0% (0.0%- 1.22%)	Na

Lower thresholds including median fluorescence intensity (MFI) and log MFI and seroprevalence with confidence intervals (CI) for Hendra virus, Cedar virus, Tioman virus and Australian bat lyssavirus for Grey-headed flying foxes sampled in Adelaide, South Australia between September 2015 and February 2018 (n = 301). na = not applicable.

### Hurdle modelling of seropositivity and antibody levels

**Fig 3:**
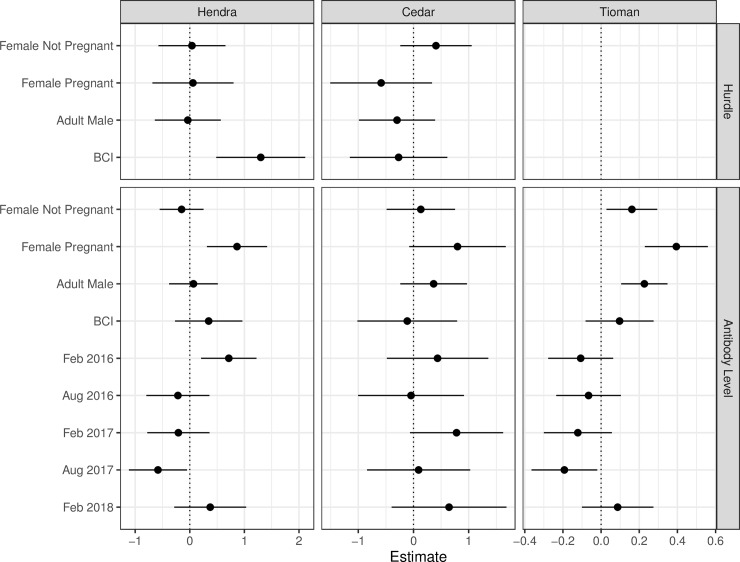
Effect plots for hurdle and antibody level models. Effect plots for the estimates and P values for negative binomial hurdle and antibody level models for Hendra virus, Cedar virus and Tioman virus serology for Grey-headed flying foxes sampled between August 2015 and February 2018.

Using lower threshold levels, the probability of Hendra virus seropositivity was positively and significantly associated with body condition index (Odds ratio = 3.67, p = 0.002). Cedar virus seropositivity was not associated with any of the investigated factors. 95.7% of all bats were Tioman virus seropositive and the hurdle model could not converge because of saturation (Table 4 and Fig 3). Using the antibody level model, Hendra virus antibody MFI levels were significantly higher in pregnant seropositive females and at the second survey in February 2016 (when 57.4% of individuals were seropositive). However, Hendra virus antibody MFI levels were significantly lower at the fifth survey in September 2017 when 37.0% of individuals were seropositive. Tioman virus MFI antibody levels were significantly higher in adults than sub-adults and at the fifth catching session in September 2017 (Table 4 and Fig 3). There was no evidence of co-seropositivity among the four viruses.

**Table 4:** Statistics for hurdle and antibody level models.

MODEL		Hendra virus					Cedar virus					Tioman virus			
** HURDLE**	**Estimate**	**OR (95% CI)**	**SE**	**Z value**	**P value**	**Estimate**	**OR (95% CI)**	**SE**	**Z value**	**P value**	**Estimate**	**OR (95% CI)**	**SE**	**Z value**	**P value**
**Intercept**	-0.311	-	0.204	-1.526	0.127	-0.996	-	0.221	-4.5	<0.001	.	.	.	.	.
**Adult male**	-0.036	-1.04	0.308	-0.118	0.906	-0.298	-1.34	0.352	-0.847	0.397	.	.	.	.	.
**Female Not Preg**	0.04	1.04	0.313	0.127	0.899	0.407	1.5	0.33	1.235	0.217	.	.	.	.	.
**Female Preg**	0.057	1.05	0.379	0.151	0.88	-0.587	-1.8	0.471	-1.246	0.213	.	.	.	.	.
**BCI centred**	**1.3**	**3.67**	**0.416**	**3.126**	**0.002**	-0.27	1.31	0.451	-0.598	0.55	.	.	.	.	.
**ANTIBODY LEVEL**	**Estimate**	**SE**	**Z value**	**P value**	**Estimate**	**SE**	**Z value**	**P value**	**Estimate**	**SE**	**Z value**	**P value**			
**Intercept**	8.717	0.218	40.027	<0.001	7.616	0.347	21.96	<0.001	7.543	0.07	108.318	<0.001			
**Adult male**	0.067	0.228	0.295	0.768	0.365	0.308	1.185	0.236	**0.227**	**0.062**	**3.667**	<0.001			
**Female Not Preg**	-0.15	0.205	-0.732	0.464	0.133	0.316	0.42	0.675	**0.161**	**0.068**	**2.374**	**0.018**			
**Female Preg**	**0.865**	**0.281**	**3.073**	**0.002**	0.797	0.446	1.787	0.074	**0.394**	**0.084**	**4.685**	**0**			
**BCI centred**	0.346	0.315	1.099	0.272	-0.113	0.461	-0.245	0.806	0.097	0.091	1.064	0.287			
**16-Feb**	**0.715**	**0.258**	**2.766**	**0.006**	0.436	0.469	0.93	0.353	-0.107	0.087	-1.231	0.218			
**16-Aug**	-0.218	0.295	-0.739	0.46	-0.045	0.489	-0.092	0.927	-0.066	0.086	-0.761	0.447			
**17-Feb**	-0.209	0.291	-0.719	0.472	0.78	0.43	1.813	0.07	-0.122	0.091	-1.341	0.18			
**17-Aug**	**-0.583**	**0.271**	**-2.152**	**0.031**	0.093	0.477	0.194	0.846	**-0.192**	**0.088**	**-2.191**	**0.028**			
**18-Feb**	0.374	0.336	1.113	0.266	0.645	0.531	1.213	0.225	0.087	0.096	0.907	0.365			

Odds ratios (OR), estimates, standard errors (SE), Z values and P values for negative binomial hurdle and antibody level models for Hendra virus, Cedar virus and Tioman virus serology for Grey-headed flying foxes sampled between August 2015 and February 2018. Reference values relate to sub-adults (both male and female) for the hurdle and antibody level model and for the first catching session (August 2015) for the antibody level model. Preg = pregnant. BCI centred = body condition index centred around the mean values. CI = confidence interval.

### Investigation of freedom from Australian bat lyssavirus seroprevalence

None of the tested bats yielded a MFI high enough to imply seroconversion. Accounting for the number of bats captured at each sampling session, there was enough evidence to demonstrate, with 95% confidence, that the Australian bat lyssavirus sero-prevalence is less that 2%, assuming that the probability of the camp to be exposed (or an immigrating bat being exposed) between samplings was ≤ 5% (Table 5). There was not enough evidence to demonstrate with confidence a seroprevalence ≤ 1% regardless of the probability of exposure.

**Table 5:** Probablity of freedom from Australian bat lyssavirus exposure.

	Probability of exposure				
Design prevalence (P*)	0.5%	1%	2%	5%	10.0%
1%	94.6%	93.8%	92.2%	87.2%	78.4%
2%	**99.5%**	**99.2%**	**98.6%**	**96.7%**	93.3%
5%	**100.0%**	**99.9%**	**99.8%**	**99.6%**	99.1%
10%	**100.0%**	**100.0%**	**100.0%**	**100.0%**	**99.9%**

Summary of final probability of freedom from Australian bat lyssavirus exposure after 6 surveys of a total 301 bats (all seronegative) from the Adelaide Grey-headed flying fox camp. Bold types represent values of probability of freedom where a minimum threshold of 95% was reached.

### Recapture seroprevalence analysis

Over the six sessions, four bats were recaught; three males and one female (Table 6). Between survey one and two, September 2015 and February 2016, respectively, the Hendra virus MFI antibody level for one male almost doubled from MFI 9428 to 16929, suggesting exposure occurred prior to September 2015 and continued until February 2016 or reinfection or recrudescence of Hendra virus occurred during this same period. This male’s weight also increased as it was classified as a sub-adult in September 2015 and an adult in February 2016. Another male seroconverted for Cedar virus between Sept 2015 and Feb 2016, suggesting exposure occurred during that period. All four animals were seropositive for Tioman virus at both sampling periods while two males did not seroconvert for Hendra virus and thus remained seronegative between the two six month time periods. Furthermore, two males and one female did not show evidence of exposure to Cedar virus between sampling periods.

**Table 6:** Seroprevalence changes in recaptured Grey-headed flying foxes.

Bat ID	Date of capture	Sex	Wt /g	BCI	Hendra virus MFI	Hendra virus seropositive	Cedar virus MFI	Cedar virus seropositive	Tioman virus MFI	Tioman virus seropositive	Australian bat lyssavirus MFI	Australian bat lyssavirus seropositive
6 & 72	31 Aug 2015	F	844	29.8	772	-	139	-	2797	+	136	-
	26 Feb 2016		763	26.9	745	-	153	-	2355	+	91	-
23 & 70	2 Sept 2015	M	820	30.1	172	-	181	-	2592	+	130	-
	26 Feb 2016		744	26.6	128	-	3073	+	1248	+	115	-
46 & 81	3 Sept 2015	M	666	25.4	9428	+	175	-	2820	+	92	-
	26 Feb 2016		773	28.0	16929	+	191	-	2979	+	141	-
145 & 252	10 Aug 2016	M	818	30.7	8710	+	257	-	2217	+	204	-
	13 Aug 2017		830	30.8	6335	+	210	-	3111	+	207	-

The sex, weight (Wt), body condition index (BCI) and study identification number (Bat ID) of four Grey-headed flying foxes recaptured between August 2015 and August 2017 at the Adelaide Camp, Adelaide, South Australia and their median fluorescence intensity (MFI) serostatus for Hendra virus, Cedar virus, Tioman virus (using lower thresholds) and Australian bat lyssavirus. M = Male; F = Female; Seropositive = +; Seronegative = —.

## Discussion

Our study showed strong evidence of exposure of Adelaide Grey-headed flying fox camp to Hendra virus, Cedar virus and Tioman virus and no evidence of exposure to Australian bat lyssavirus. The semiquantitative results provided by Luminex binding assays also identified individual-level correlates of seroposivity and antibody levels. Hendra virus seroprevalence in this study (43.2%, 95%CI: 37.5%-49%) is similar to that reported previously (44.5%) [31] using a Luminex binding assay and compares with an overall seroprevalence of 23.6% using a serum neutralisation test in Little red flying foxes (*P*.*scapulatus*) [23] and an overall seroprevalence of 56% using a serum neutralisation test in Spectacled flying foxes [21]. Cedar virus seroprevalence was half than previously reported; 26.6% (95%: 21.7%-31.9%) versus 51.1% in Grey-headed flying foxes [31]. There were some differences in the exposure rates within the camp and across the study sampling times.

Hendra seropositivity was also positively associated with BCI. This contrasts with a previous study [23] which found increased seropositivity in nutritionally-stressed Little-red flying foxes but concurs with a study [10] in Black flying foxes and Grey-headed flying foxes. In our study, the body condition index of bats was significantly higher in winter than summer (Table 2). Food quantity and quality for Grey-headed flying foxes are usually inferior in winter elsewhere in their normal range [50]. Winter immigration of approximately 5–10,000 extra Grey-headed flying foxes into the Adelaide camp (Van Weenan pers comm) in 2018 and 2019 suggests that Adelaide is an attractive feeding ground during winter. Other studies indicate acute food shortages may be associated with El Nino/La Nina climate cycles [51] leading to nutritionally stressed animals and this may be the driver for the seasonal patterns of Hendra virus seroprevalence [23, 52]. Late gestation was positively associated with higher Hendra virus MFI antibody levels in comparison to non-pregnant females and males. SImilar evidence is seen in serological surveys of Spectacled flying foxes [21] and Little-red flying foxes [23] which showed increased detection of Hendra virus antibodies associated with late-stage gestation or early lactation but is in contrast to recent research in Grey-headed flying foxes [10] where there is no association.

Hendra virus and Tioman virus seropositivity varied across surveys (Fig 3) with Hendra virus seroprevalence significantly increasing between August 2015 and February 2016. This pattern could be explained by: i) “exposure and spread in a sedentary camp” where a Hendra virus exposure event that occurred before August 2015 (seroprevalence = 49%) and resulted in an increase in seroprevalence of captured animals in February 2016 (seroprevalence = 57%) without any further exposure occurring in this period (i.e. within camp spread) and negligible emigration/immigration; or ii) “exposure and re-exposure in a sedentary camp” where additional Hendra virus exposure occurred between the two sampling periods which led to an higher seroprevalence at the second sampling period and negligible emigration/immigration; or iii) “periodic emigration” of non-exposed animals and/or “periodic immigration” of previously exposed flying foxes occurred during this period. Previous studies have suggested that Hendra virus is maintained in flying fox populations through episodic infection in a metapopulation structure, and do not persist endemically within a single population [23]. Most hypotheses emphasize horizontal transmission within colonies via urine and other secretions, especially during pregnancy and mating [23], or via migration, with the magnitude of migration affected by the spatial connectivity among colonies, resulting in episodic infection [53].

The recaptures of four individuals over the sampling period provided some information on the immunity dynamics of these viruses within this specis. Two of these animals were not exposed to Hendra virus and thus remained seronegative between the two captures (six month time period for both). However, one animal’s Hendra virus seropositive MFI antibody level nearly doubled over a six month time period; between September 2015 and February 2016 which could mean: i) it was recently exposed just before the first sampling and the antibody level continued to rise in response to the second sampling; ii) it was exposed some time before the first sampling and the antibody level peaked between the two captures and was waning at the second; or iii) it was exposed some time before the first sampling and was re-exposed between captures and mounted a further antibody response. Epstein et al, 2013 [30] suggests maternal antibodies to Hendra virus in Black flying foxes last between 7.5 and 8.5 months and acquired immunity to African henipaviruses may last up to 4 years in adult *Eidolon helvum* adults [54] but evidence on Grey-headed flying foxes immune response to viruses is sparse. The antibody level of another seropositive animal waned over one year between August 2016 and 2017, suggesting that the animal was less likely to have become further infected (August 2016 to August 2017). The fourth animal was seropositive and its antibody level waned over one year (August 2016 to August 2017), suggesting that this animal was unlikely re-exposed during this period.

None of the explored explanatory variable predicted Cedar virus serostatus which is consistent with previous reports [31]. Furthermore there was no evidence of association between the serostatuses of any pair of viruses. Adult bats showed significantly higher antibody levels against Tioman virus in comparision to subadults which may suggest there is a cumulative age-related antibody response to multiple exposures of the virus. Additionally, immunofluorescent antibody and immunoelectron microscopic data suggested that Tioman virus is antigenically related to Menangle virus [12] so it is possible that the high seropositivity to Tioman virus could result from the cross reactivity with Menangle virus exposure.

No evidence of Australian bat lyssavirus exposure was found over our study period despite a previous finding of a positive diagnosis in a Grey-headed flying fox in the Adelaide camp in September, 2012 [20]. Previous serological surveys have found a 3.0% Australian bat lyssavirus seroprevalence in flying foxes (95% CI: 1.5–5.8%)[55] using the rapid fluorescent focus inhibition test and 2.9% seroprevalence (95% CI: 1.8–4.5%) in six insectivorous species in Western Australia using a Luminex multiplex binding assay [33]. Rabies virus neutralising antibodies have been shown to wane in experimentally-infected bats within 6 months after an initial inoculation, but persisted for longer (6–12 months) after a second inoculation of surviving bats [56]. Our results suggest that either (i) Australian bat lyssavirus has not been circulating in the camp over this time period; (ii) seropositivity is very short lived; or (iii) infected flying foxes died suddenly and were thus not sampled at surveys. However, bats are thought to be the ancestral reservoir of lyssaviruses [57] and are the only taxa in which antibodies are detected with sufficient frequency to support serosurveillance [33] which could indicate that the virus is unlikely to be circulating in the Adelaide camp.

As with all flying fox camps, the population dynamics can often be very fluid with regular patterns of immigration, emigration and range expansions. Some studies show flying foxes can travel hundreds of kilometres [15, 55, 58], moving regularly between different camps over their distribution range. Furthermore, there is evidence that all four species of mainland Australian pteropids can co-occur in the same camp [59]. Range expansions and contractions have been noted in both Black flying foxes and Grey-headed flying foxes [18, 60–61]. The range of Black flying foxes has increased southwards greater than 1000km during the twentieth century [62] and this has been proposed as a possible contributing factor to a contraction of Grey-headed flying foxes distribution range. In its southernmost distribution, Grey-headed flying foxes now live in the urban environments of Melbourne [61] and Adelaide. While these areas are not thought to be part of the ‘climatic niche’ of the species during winter, increased temperature due to the ‘urban heat island effect’ and climate change may have created an environment that is now tolerable [63]. Therefore its conceivable that through this overlap of flying fox species, transmission and infection may occur anywhere along the distribution range continuum at any time.

Microsphere assays provide a sensitive method to detect henipavirus antibody binding in fruit bat plasma and serum [15, 32, 36].The output of these assays, median fluorescence intensity (MFI), are continuous data and present a challenge in determining meaningful threshold values that categorise bats as seropositive or seronegative [36]. A MFI > 1,000 for Australian bat lyssavirus has been considered positive and < 250 negative [33]. Our use of mixture models to determine threshold values reflects that of Burroughs et al 2016 [31] in that we accept that a single threshold is not possible for the serological profile obtained for the Adelaide bats. We looked for ‘natural’ groupings of binding activity and used two threshold values to divide these groups into negative, intermediate and positive categories. We recognise that binding in the intermediate category may represent an important intermediate stage in antiviral protection, the shift from a seronegative to a seropositive state or vice versa, or may represent a susceptible state. Even using the more specific threshold (MFI 5825, 1702, 1603 for Hendra virus, Cedar virus and Tioman virus, respectively), 25.2% of bats caught from the Adelaide camp showed evidence of prior infection with Hendra virus, 16.6% with Cedar virus and 63.8% with Tioman virus which all suggest common exposures at both the individual and camp level.

We acknowledge certain limitations to our study. The most effective technique to capture bats in the Adelaide camp requires nets to be placed from suitable trees under the camp as they return from foraging. The entire foot print of bat roost trees could not be sampled using a formal random sampling approach because of the topography and may therefore consistute a potential sampling bias. Utilising the same capture sites across the whole study period attempted to standardise any potential sampling bias and protect the comparibility of samples.

## Conclusion

In contrast to other studies, good body condition rather than nutritional stress was an indicator of increased Hendra virus seroconversion. Substantiating other studies, Hendra virus antibody levels were higher in pregnant females. Unexpectedly, there was no evidence of Australian bat lyssavirus seroconversion. This study highlighted the successful use of a multiplexed Luminex binding assay for serological surveys in flying foxes but also the need to expand the research to include more sampling periods over an annual cycle and to compare with viral presence and diversity.

## Supporting information

S1 Table Catching information, demographic data, morphometrics and MFI values for Hendra virus, Cedar virus, Tioman virus and Australian bat lyssa virus for each sampled bat. (XLSX)

## References

[1] Jones KE, Patel NG, Levy MA, Storeygard A, Balk D, Gittleman JL et al Global trends in emerging infectious diseases. Nature. 2008; 451: 990–993. 10.1038/nature06536 18288193 PMC5960580

[2] Calisher CH, Childs JE, Field HE, Holmes KV, Schountz T. Clin Microbiol Rev. 2006; 19: 531–545. 10.1128/CMR.00017-06 16847084 PMC1539106

[3] Halpin K, Hyatt AD, Fogarty R, Middleton D, Bingham J, Epstein JH et al Pteropid bats are confirmed as the reservoir hosts of henipaviruses: a comprehensive experimental study of virus transmission. Am J Trop Med Hyg. 2011; 85: 946–951. 10.4269/ajtmh.2011.10-0567 22049055 PMC3205647

[4] Smith I and Wang L-F. Bats and their virome. An importance source of emerging viruses capable of infecting humans. Curr Opin Virol. 2013; 3: 84–91. 10.1016/j.coviro.2012.11.006 23265969 PMC7102720

[5] Wong ACP, Li X, Lau SKP, Woo PCY. Global Epidemiology of Bat Coronaviruses. Viruses. 2019; 11: 174 10.3390/v11020174 30791586 PMC6409556

[6] Fraser GC, Hooper PT, Lunt RA, Gould AR, Gleeson LJ, Hyatt AD et al Encephalitis caused by a lyssavirus in fruit bats in Australia. Emerg Infect Dis. 1996; 2: 327–31. 10.3201/eid0204.960408 8969249 PMC2639915

[7] Philbey AW, Kirkland PD, Ross AD, Field HE, Srivastava M, Davis RJ et al Infection with Menangle virus in flying foxes (*Pteropus* spp.) in Australia. Aust Vet J. 2008; 86: 449–454. 10.1111/j.1751-0813.2008.00361.x 18959537

[8] Field H, de Jong C, Melville D, Smith C, Smith I, Broos A et al Hendra Virus Infection Dynamics in Australian Fruit Bats. PLoS One. 2011; 6 (12): e28678 10.1371/journal.pone.0028678 22174865 PMC3235146

[9] Barr JA, Smith C, Marsh GA, Field H, Wang L-F. Evidence of bat origin for Menangle virus, a zoonotic paramyxovirus first isolated from diseased pigs. J Gen Virol. 2012; 93: 2590–4. 10.1099/vir.0.045385-0 22915696

[10] Edson D, Peel AJ, Huth L, Mayer DG, Vidgen ME, McMichael L et al Time of year, age class and body condition predict Hendra virus infection in Australian black flying foxes (*Pteropus alecto*). Epid and Infection. 2019; 10.1017/S0950268819001237 PMC6625375 31364577

[11] Barr J, Smith C, Smith I, de Jong C, Todd S, Melville D et al Isolation of multiple novel paramyxoviruses from pteropid bat urine. J Gen Virol. 2015; 96: 24–29. 10.1099/vir.0.068106-0 25228492

[12] Chua KB, Wang LF, Lam SK, Crameri G, Yu M, Wise T. Tioman virus, a novel paramyxovirus isolated from fruit bats in Malaysia. Virology. 2001; 283: 215–229. 10.1006/viro.2000.0882 11336547

[13] Yaiw KC, Bingham J, Crameri G, Mungall B, Hyatt A, Yu M et al Tioman Virus, a Paramyxovirus of Bat Origin, Causes Mild Disease in Pigs and Has a Predilection for Lymphoid Tissues. J Virol. 2007; 82: 565–568; 10.1128/JVI.01660-07 17913804 PMC2224375

[14] Threatened Species Scientific Committee (2001). *Commonwealth Listing Advice on* Pteropus poliocephalus (*Grey-headed Flying-fox*). Available from: http://www.environment.gov.au/biodiversity/threatened/species/p-poliocephalus.html. In effect under the EPBC Act from 06-Dec-2001. Accessed 6 November 2019.

[15] Breed AC, Yu M, Barr JA, Crameri G, Thalmann CM, Wang LF. Prevalence of henipavirus and rubulavirus antibodies in pteropid bats, Papua New Guinea. Emerg Infect Dis. 2010; 16: 1997–1999. 10.3201/eid1612.100879 21122242 PMC3294587

[16] Kessler MK, Becker DJ, Peel AJ, Justice NV, Lunn T, Crowley DE et al Changing resource landscapeds and spillover of henipaviruses. Ann NY Acad Sci. 2018; 1429 78–99 10.1111/nyas.13910 PMC6778453 30138535

[17] Eby P, Richards G, Collins L, Parry-Jones K. The distribution abundance and vulnerability to population reduction of a nomadic nectarivore, the grey-headed flying-fox Pteropus poliocephalus in New South Wales, during a period of resource concentration. Aust. Zool. 1999; 31: 240–253. 10.7882/AZ.1999.024

[18] Williams NSG, McDonnell MJ, Phelan GK, Keim L D and Van der Ree R. Range expansion due to urbanization: increased food resources attract grey-headed flyingfoxes (*Pteropus poliocephalus*) to Melbourne. Austral Ecol. 2006; 31: 190–198. 10.1111/j.1442-9993.2006.01590.x

[19] Tait J, Perotto-Baldivieso HL, McKeown A, Westcott DA. Are Flying-Foxes Coming to Town? Urbanisation of the Spectacled Flying-Fox (*Pteropus conspicillatus*) in Australia. PLoS One. 2014; 9 (10): e109810 10.1371/journal.pone.0109810 25295724 PMC4190360

[20] Cox-Witten K. Australian bat lyssavirus Bat Stats. Wildlife Health Australia website. Sourced November 2019. Available from https://www.wildlifehealthaustralia.com.au/Portals/0/Documents/ProgramProjects/ABLV_Bat_Stats_Jun_2019.pdf

[21] Breed AC, Breed MF, Meers J, Field HE. Evidence of Endemic Hendra Virus Infection in Flying-Foxes (*Pteropus conspicillatus*)—Implications for Disease Risk Management. PLoS One. 2011; 6 (12): e28816 10.1371/journal.pone.0028816 22194920 PMC3237542

[22] Hayman DTS, Suu-Ire R, Breed AC, McEachern JA, Wang L, Wood JLN, et al Evidence of henipavirus infection in west African fruit bats. PLoS One. 2008; 3 (7): e 2739 10.1371/journal.pone.0002739 PMC2453319 18648649

[23] Plowright RK, Field HE, Smith C, Divljan A, Palmer C, Tabor G et al Reproduction and nutritional stress are risk factors for Hendra virus infection in little red flying foxes (*Pteropus scapulatus*). Proc R Soc Lond Biol. 2008; 275: 861–869. 10.1098/rspb.2007.1260 PMC2596896 18198149

[24] Pourrut X, Delicat A, Rollin PE, Ksiazek TG, Gonzalez JP, Leroy EM. Spatial and temporal patterns of Zaire ebolavirus antibody prevalence in the possible reservoir bat species. J Infect Dis. 2007; 196 S176–S183. 10.1086/520541 17940947

[25] Plowright RK, Peel AJ, Streicker DG, Gilbert AT, McCallum H, Wood J et al Transmission or Within-Host Dynamics Driving Pulses of Zoonotic Viruses in Reservoir-Host Populations. PLoS Negl Trop. Dis. 2016; 10 (8): e0004796 10.1371/journal.pntd.0004796 27489944 PMC4973921

[26] Epstein JH, Prakash V, Smith CS, Daszak P, McLaughlin AB, Meehan G et al Henipavirus infection in fruit bats (Pteropus giganteus), India. Emerg Infect Dis. 2008; 14: 1309–1311. 10.3201/eid1408.071492 18680665 PMC2600370

[27] Gilbert A, Fooks AR, Hayman DTS, Horton DL, Müller T, Plowright R et al Deciphering serology to understand the ecology of infectious diseases in wildlife. Ecohealth. 2013; 10: 298–313. 10.1007/s10393-013-0856-0 23918033

[28] Daniels P, Ksiazek T, Eaton BT. Laboratory diagnosis of Nipah and Hendra virus infections. Microbes Infect. 2001; 3: 289–295. 10.1016/s1286-4579(01)01382-x 11334746

[29] Bossart KN, McEachern JA, Hickey AC, Choudhry V, Dimitrov DS, Eaton BT et al Neutralization assays for differential henipavirus serology using Bio-Plex Protein array systems. J Virol Methods. 2007; 142: 29–40. 10.1016/j.jviromet.2007.01.003 17292974

[30] Epstein JH, Baker ML, Zambrana-Torrelio C, Middleton D, Barr JA, Dubovi E, et al Duration of maternal antibodies against canine distemper virus and Hendra virus in Pteropid bats. PLoS One.2013; 8(6): e67584 10.1371/journal.pone.0067584 23826322 PMC3695084

[31] Burroughs AL, Durr PA, Boyd V, Graham K, White JR, Todd S et al Hendra virus infection dynamics in the grey-headed flying fox (Pteropus poliocephalus) at the southern-most extent of Its range: further evidence this species does not readily transmit the virus to horses. PLoS ONE. 2016; 11 (6): e0155252 10.1371/journal.pone.0155252 27304985 PMC4909227

[32] McNabb L, Barr J, Crameri G, Jusva S, Colling A, Riddell S et al Henipavirus microsphere immune-assays for detection of antibodies against Hendra virus. J Virol Methods. 2014; 200: 22–28. 10.1016/j.jviromet.2014.01.010 24508193 PMC8846554

[33] Prada D, Boyd V, Baker M, Jackson B, O’Dea M. Insights into Australian Bat Lyssavirus in Insectivorous Bats of Western Australia. Trop Med Infect Dis. 2019; 4: 46 10.3390/tropicalmed4010046 PMC6473685 30862028

[34] Hayman DTS, Wang LF, Barr J, Baker KS, Suu-Ire R, Broder CC et al Antibodies to henipavirus or henipa-like viruses in domestic pigs in ghana, West Africa. PLoS One. 2011; 6 10.1371/journal.pone.0025256 PMC3178620 21966471

[35] Peel AJ, Bake KS, Crameri G, Barr JA, Hayman DTS, Wright E et al Henipavirus neutralising antibodies in an isolated island population of African fruit bats. PLoS One. 2012; 7 (1): e30346 10.1371/journal.pone.0030346 22253928 PMC3257271

[36] Peel AJ, McKinley TJ, Baker KS, Barr JA, Crameri G, Hayman DTS et al Use of cross-reactive serological assays for detecting novel pathogens in wildlife: Assessing an appropriate cutoff for henipavirus assays in African bats. J Virol Methods. 2013; 193: 295–303. 10.1016/j.jviromet.2013.06.030 23835034 PMC8796701

[37] Sendow I, Ratnawati A, Taylor T, Adjid RM, Saepulloh M, Barr J et al Nipah virus in the fruit bat Pteropus vampyrus in Sumatera, Indonesia. PLoS One. 2013; 8 (7): e69544 10.1371/journal.pone.0069544 23894501 PMC3718695

[38] Edson D, Field H, McMichael L, Vidgen M, Goldspink L, Broos A et al Routes of Hendra virus excretion in naturally infected flying-foxes: implications for viral transmission and spillover risk. PLoS One. 2015; 10 (10): e0140670 10.1371/journal.pone.0140670 26469523 PMC4607162

[39] Peel AJ, Wells K, Giles J, Boyd V, Burroughs A, Edson D et al Synchronous shedding of multiple paramyxoviruses coincides with peak periods of Hendra virus spillover. Emerg Micr Infect. 2019; 8: 1314–1323, 10.1080/22221751.2019.1661217 31495335 PMC6746281

[40] Hall L, Richards G. Flying foxes: fruit and blossom bats of Australia. 2000 Dawson TJ, ed. Sydney: University of New South Wales Press Ltd.

[41] Williamson MM, Hooper PT, Selleck PW, Westbury HA, Slocombe RF. Experimental Hendra virus infection in pregnant guinea-pigs and fruit bats (*Pteropus poliocephalus*). J Comp Pathol. 1999; 122201–7. 10.1053/jcpa.1999.0364 10684689

[42] Halpin K, Young PL, Field HE, McKenzie JS. Isolation of Hendra virus from pteropid bats: a natural reservoir of Hendra virus. J Gen Virol. 2008; 1: 1927–1932. 10.1099/0022-1317-81-8-1927 10900029

[43] Horby PW, Laurie KL, Cowling BJ, Engelhardt OG, Sturm‐Ramirez K, Sanchez JL et al CONSISE statement on the reporting of Seroepidemiologic Studies for influenza (ROSES‐I statement): an extension of the STROBE statement. Influenza Other Respir Viruses. 2017; 11: 2–14. 10.1111/irv.12411 27417916 PMC5155648

[44] Jonsson NN, Johnston SD, Field H, de Jong C, Smith C. Field anaesthesia of three Australian species of flying fox. Vet Rec. 2004; 154: 664 10.1136/vr.154.21.664 15198316

[45] Schulz JE, Seifert SN, Thompson JT, Avanzato V, Sterling SL, Yan L et al Serological Evidence for Henipa-like and Filo-like Viruses in Trinidad Bats. J Infect Dis. 2020 jiz648 10.1093/infdis/jiz648 32034942 PMC7213578

[46] Laing E, Yan L, Sterling S, Broder C. A Luminex-based multiplex assay for the simultaneous detection of glycoprotein specific antibodies to ebolaviruses, marburgviruses, and henipaviruses. 2016; Int J Infect Dis. 53, Supplement, 108–109. 10.1016/j.ijid.2016.11.272

[47] Petraityte R, Tamosiunas PL, Juozapaitis M, Zvirbliene A, Sasnauskas K, Shiell B, et al Generation of Tioman virus nucleocapsid-like particles in yeast *Saccharomyces cerevisiae*. Virus Res. 2009: 145:92–96. 10.1016/j.virusres.2009.06.013 19559738

[48] Rahmadane I, Certoma AF, Peck GR, Fitria Y, Payne J, Colling A, et al Development and validation of an immunoperoxidase antigen detection test for improved diagnosis of rabies in Indonesia. PLoS Negl Trop Dis. 2017; 11(11): e0006079 10.1371/journal.pntd.0006079 29131859 PMC5703572

[49] Cameron A, Njeumi F, Chibeu D. and Martin T. Risk-based disease surveillance. Food and Agriculture Organization of the United Nations; 2015.

[50] Eby P and Law B. Ranking the feeding habitat of grey-headed flying foxes for conservation management. http://www.environment.nsw.gov.au/resources/threatenedspecies/GHFFmainreport.pdf. 2008 Last accessed on November 5, 2019. 10.1371/journal.pone.0067584 . 23826322 PMC3695084

[51] Giles JR, Plowright RK, Eby P, Peel AJ, McCallum H. Models of Eucalypt phenology predict bat population flux. Ecol Evol. 2016; 6: 7230–7245. 10.1002/ece3.2382 27891217 PMC5115174

[52] Plowright RK, Eby P, Hudson PJ, Smith IL, Westcott D, Bryden WL, et al Ecological dynamics of emerging bat virus spillover. Proc R Soc Lond Biol. 2015; 282: 2014–2124. 10.1098/rspb.2014.2124 PMC4262174 25392474

[53] Wang HH, Kung NY, Grant WE, Scanlan JC, Field HE. Recrudescent infection supports Hendra virus persistence in Australian flying-fox populations. PLoS One. 2013; 8: 10.1371/journal.pone.0080430 PMC3842926 24312221

[54] Peel AJ, Baker KS, Hayman DTS, Broder CC, Cuningham AA, Fooks AR et al Support for viral persistence from age-specific serology and models of immunity. Nat. Sci. Rep. 2018; 10.1038/s41598-018-22236-6 29497106 PMC5832774

[55] Field HE. Evidence of Australian bat lyssavirus in diverse Australian bat taxa. Zoonoses Public Health. 2018; 65: 742–748 10.1111/zph.12480 29785730 PMC6249124

[56] Turmelle AS, Jackson FR, Green D, McCracken GF, Rupprecht CE. Host immunity to repeated rabies virus infection in big brown bats. J Gen Virol. 2010; 91: 2360–2366. 10.1099/vir.0.020073-0 20519458 PMC3052523

[57] Badrane H and Tordo N. Host Switching in *Lyssavirus* History from the Chiroptera to the Carnivora Orders. J Virol. 2001; 17: 8096–8104. 10.1128/JVI.75.17.8096-8104.2001 PMC115054 11483755

[58] Tidemann CR and Nelson JE. Long-distance movements of the grey-headed flying fox (*Pteropus poliocephalus*). J Zool. 2004; 263: 141–146. 10.1017/S0952836904004960

[59] Parsons JG, Van Der Wal J, Robson SKA, Shilton LA. The implications of sympatry in the spectacled and grey headed flying-fox, *Pteropus conspicillatus* and *P*. *poliocephalus* (Chiroptera: Pteropodidae). Acta Chirop. 2010; 12: 301–309. 10.3161/150811010X537882

[60] Tidemann CR. Biology and management of the Grey-headed Flying-fox, *P*. *poliocephalus*. Acta Chirop. 1999; 1: 151–64

[61] van der Ree R, McDonnell MJ, Temby ID, Nelson J. and Whittingham E. The establishment and dynamics of a recently established urban camp of (*Pteropus poliocephalus*) outside their geographic range. J. Zool. 2006; 268: 177–85. 10.1111/j.1469-7998.2005.00005.x

[62] Roberts BJ, Catterall CP, Eby P, Kanowski J. Latitudinal range shifts in Australian flying-foxes: a re-evaluation. Austral Ecol. 2012; 37: 12–22. 10.1111/j.1442-9993.2011.02243.x

[63] Parris KM and Hazell DL. Biotic effects of climate change in urban environments: the case of the Grey-headedFlying-fox (*Pteropus poliocephalus*) in Melbourne. Biol.Conserv. 2005; 124: 267–76. 10.1016/j.biocon.2005.01.035

