# Supplementary material for: Seroprevalence of three paramyxoviruses; Hendra virus, Tioman virus, Cedar virus and a rhabdovirus, Australian bat lyssavirus, in a range expanding fruit bat, the Grey-headed flying fox (Pteropus poliocephalus)
Source: PLoS One. 2020 May 6;15(5):e0232339. doi: 10.1371/journal.pone.0232339 (PMC7202650; doi:10.1371/journal.pone.0232339)
Supplement: Attachment — Submitted filename: BOARDMAN Rebuttal Letter_V03.docx [file pone.0232339.s002.docx]

**Rebuttal Letter**

**General Comments**

| Please ensure that your manuscript meets PLOS ONE's style requirements, including those for file naming. The PLOS ONE style templates can be found at  <http://www.journals.plos.org/plosone/s/file?id=wjVg/PLOSOne_formatting_sample_main_body.pdf>  <http://www.journals.plos.org/plosone/s/file?id=ba62/PLOSOne_formatting_sample_title_authors_affiliations.pdf> | We have reviewed this and believed we have covered everything required and made changes accordingly. We have altered the co-authors slightly and added further acknowledgements |
| --- | --- |
| We note that you have reported significance probabilities of 0 in places. Since p=0 is not strictly possible, please correct this to a more appropriate limit, eg 'p<0.0001' | We have changed this throughout |
| We note that [Figure(s) 1] in your submission contain [map/satellite] images which may be copyrighted. All PLOS content is published under the Creative Commons Attribution License (CC BY 4.0), which means that the manuscript, images, and Supporting Information files will be freely available online, and any third party is permitted to access, download, copy, distribute, and use these materials in any way, even commercially, with proper attribution. For these reasons, we cannot publish previously copyrighted maps or satellite images created using proprietary data, such as Google software (Google Maps, Street View, and Earth). For more information, see our copyright guidelines: <http://journals.plos.org/plosone/s/licenses-and-copyright>. | This has been changed and we have used OpenStreetMap which available under the Open Database Licence at www.openstreetmap.org/copyright. |
| In your Methods section, please provide additional location information, including geographic coordinates for the data set if available. | Geographic coordinates for the camp have been added to the text. 34°54'56 S, 138°36'24 E |

**Reviewer 1 - Major Revisions – OLD LINES	NEW LINES IN CLEAN DOCUMENT**

| I believe that the introduction and discussion can be streamlined. The introduction is 8 paragraphs and the discussion is 9 paragraphs. There is much salient data in these sections, but they should be cut down for the benefit of the reader. | Thanks for the comment – we believe we have streamlined the content now – we hope what we have done is acceptable. |
| --- | --- |
| The methods section for the serology, which plays an integral role in the study results, is not detailed enough. The authors should provide a significantly more detailed section including; the proteins (target and how they are produced, if samples were run in duplicate/triplicate, what machine they were run on. I understand that the authors are referencing a previous study, but I imagine details in the bat handling and serum collection could be similarly truncated with a reference to similar approaches. Additionally, reference 31 in this section is for nucleic acid detection and not antibody detection and needs to be removed. | We have made changes and added the following to the text.  Briefly, the assay proteins were coupled to individual microsphere bead sets, of predetermined numbers of magnetic beads MagPlex®. These were added to each well and then mixed with bat sera at a dilution of 1:50. The bound antibody was detected using biotinylated Protein A (Pierce, Rockford, USA) together with biotinylated Protein G (Pierce, Rockford, USA) followed by streptavidin–phycoerythrin (Qiagen Pty Ltd, Australia). The assay was read using a Bio-Plex Protein 200 Array System integrated with Bio-Plex Manager Software (v 6.2) (Bio Rad Laboratories, Inc., CA, USA) calibrated on the high setting. Each sample was tested in a well with thousands of beads and the fluorescence results of 100 MagPlex® beads were recorded as the median florescent intensity (MFI) that excludes outliers and are correlated to antibody concentration  Reference 31 removed. |
| Have the authors looked at the significance of using weight only as opposed to using body condition index when studying seroprevalence. A meta-analysis done by McGuire et al 2018 (<https://doi.org/10.1093/jmammal/gyy103>) found that there was no support that, “bats with longer forearms weigh more than bats with shorter forearms”. They note that the relationship between body mass and forearm length was very weak. Is this the case in Pteropus poliocephalus and is BCI an appropriate metric to study? | McGuire et al. studied 5 species of insectivore bats (assumed microbats?) from North America and mainly grown-up individuals and non-pregnant (“*To avoid confounding effects of pregnancy or continued somatic growth of young bats, pregnant and newly volant bats were excluded*.”) therefore they had a very homogenous group of individuals within each species where FAL did not varied much and therefore not much correlation with BW. See picture below where each species cloud is roughly round and not going much upward.  The story is different for us because i) very different morphology of our bat species; ii) covering all strata of the demography (including young and pregnant).  The range of forearm lengths (FAL) measured in our study population ranged from 127.3 to 176.8 mm with a mean of 160.0. This suggests substantial variability in our FAL data (coefficient of variation = SD/mean = 90.7%) which needed to be accounted for when comparing the body condition of different sized bats. We did notice that the body condition index (BCI) was mainly driven by body weight but comparison across individuals seemed more objective when FAL is taken into consideration. We therefore maintain this approach even if FAL has less influence. |
| The only reference to raw MFI values was in the recapture data. There was no supplementary table with the MFI values. The authors should include this data and it would help if they added a scatterplot or box plot with these values for each virus glycoprotein with the lower and upper thresholds noted. Or they can add a secondary x-axis on figure 2 to know what the MFI values are. This is important because these multiplex serological assays are being implemented on pteropodid bats across South and Southeast Asia and it’s important for comparative studies to know what raw MFI values are for seropositives and seronegatives. | Thanks for these comments – we have added the supplementary table with MFI values as S1 Supporting information.xls  We also made changes by adding a secondary x axis on Figure 2. |
| The authors should be cautious when they use the word antibody titres in the text and in the methods and results section headers. They state that antibody titre is expressed as MFI, but titre has a specific meaning. Have the authors run an endpoint dilution to then correlate that endpoint with MFI detection values? If they have not, then they should not use the word titre.- | We agree and apologise for this oversight.  We have now used MFI levels or MFI antibody levels as MFI levels are not a direct comparison to titre and changed throughout. |

**Reviewer 1 - Minor Revisions – OLD LINES	NEW LINES IN CLEAN DOCUMENT**

| Line 37: What does ‘camp antibody’ titres mean? | Removed the word ‘camp’ |
| --- | --- |
| Lines 52&53: Check duplicate references | Duplicate references removed |
| Line 72: Is there a reference for the range expansion of the species? | There is no definitive reference for this but we were there when it happened and monitored the population ever since |
| Line 82: Please use another word for elusive | Thanks – changed to restricted |
| Line 88-89: The authors should mention the conformational state of the proteins in these tests as native state antigens will represent what the immune systems sees compared to linear peptides. | Instead of adding information here about the conformational state of the proteins because it is the introduction, we have added this information to the materials and methods section instead where we feel it is more appropriate. We added at CLEAN COPY Lines 169-173 the following:  “The conformational status of the viruses used were the following; soluble native-like oligomeric G envelope glycoproteins of HeV and CedV (sG_tet_) were produced from stable expressing FreeStyle^TM^ 293F cell lines  [45,46], Tioman virus was a nucleocapsid protein expressed in the yeast *Saccharomyces cerevisiae* [47], and Australian bat lyssavirus was a nucleocapsid protein prepared in *E.coli* [48].” |
| Line 97: The authors use both recrudescence and reactivation in this manuscript. Please use one or the other for consistency. | Thanks for the comment - We have used reactivation throughout |
| Line 144: What is the company that makes the mist nets? | Ecotone – added to the manuscript |
| Is estimated age a subjective categorical variable? Please explain. | Thanks – we have adjusted the text to reflect this omission. We have used Hall and Richards as a reference to age flying foxes which is now included in the text. |
| How was the sera treated before the test? Was it heat inactivated? Was it gamma inactivated? | The following has been added to the text. CLEAN COPY 173-174  Prior to analyses, serum samples were first heat treated at 56°C for 30 minutes to inactivate complement. |
| Line 169: Was the concentration of antibodies measured? It is better to state that antibodies were detected against the different viruses in the panel | Agreed – we have changed to detected and reflected this in the manuscript |
| Line 172: Please remove reference 31 as it does not refer to antibodies | Removed |
| Line 183 section: Were any priors used for Hendra and Cedar virus when running the models? There were priors used and noted for Australian bat lyssavirus, but none are mentioned for these two henipaviruses, even though there are previous studies studying seroprevalence. Noted in line 339-343 | The finite mixture and hurdle modelling were not Bayesian analyses and, therefore, no priors were required. |
| Line 196: Can you please explain how threshold values were ‘visually determined’? | Threshold values were selected as the MFI (log scaled) value for which the predicted normal distribution of two sub-population intersect. This is visually represented in Figure 2 and was further explained in the text |
| In the demographic section, some description of the collections would be helpful (adults compared to sub adults and males compared to females). I understand this is noted in the table, but some more detail in the text would be helpful. | Basic demographic descriptions were added to the section. CLEAN COPY Lines 236-242. |
| The rest of the results reads well. | Thanks |
| Are there specific periods of birthing in this species? There is a lot of natural history information, but I did not note this important fact. | Thanks – we have added this to the manuscript – CLEAN COPY line 109-110 |
| Line 376 The authors note that their findings support the SIRS and SILI models. Can they expand on this statement in this paragraph. | We have added some information to the manuscript which we hope makes it easier to understand. |
| Line 401: Why wasn’t there an association between co-serostatus? | None of the surveyed bat were seropositive to Australian bat lyssavirus, so it could not be associated with the serostatus of any of the other viruses. Similarly, most (95%) surveyed bats were seropositive to Tioman virus, so it is very unlikely to find an association with other viruses’ serostatus. Finally, 50% (40/80) of the Cedar virus seropositives and 40.7% (90/221) of the seronegatives were seropositive to Hendra virus. This resulted into a Risk Ratio of 1.23 (95%: 0.937-1.61) which is not-significantly different from 1.00 (P=0.151). Therefore, according to our study data, we found that the serostatus of one virus did not predict the serostatus of any other virus suggesting no association between serostatuses. |
| Line 413: The authors can reference Peel et al’s work on Africa henipavirus immunity duration (DOI: 10.1038/s41598-018-22236-6) and Schuh et al 2019 (Comparative analysis of serologic cross-reactivity using convalescent sera from filovirus-experimentally infected fruit bats) and this would support their discussion on cross-reactivity and decay in filoviruses. | Thanks – sorry about the omission. We have included Peels et al reference and decided to leave Schuh et al’s paper out as it relates to filoviruses which is not the subject of this paper |
| Line 438-439: There are references to specific MFI values, but there are for both henipaviruses and lyssaviruses. This sentence needs to be more detailed as to which MFI values correspond to what specific viruses. | Thanks- we have clarified in the text |
| Line 446: The author’s reference the specific thresholds for MFI values and having an additional figure with the non-transformed MFI values would help. | We have added this information to Fig 2 |
| Line 466-467: The authors state that there is no significant co-occurrence of the studied viruses, but they did not test for viruses. They only studied seroprevalence. | Changed to:  There was no significant antibody co-serostatus of the studied viruses |
| Table 2  It would be beneficial to have the range of MFI values and the median values for seropositives in this table | Thanks – these values have been added to Table 2 |
| Figures Figure 1 looks to be from google maps. That satellite image from a copyrighted source may not be publishable under the PLOS Open Access CC-BY License | Thanks – we have made a more detailed map which is now open access |

**Reviewer 2 - Major comments – OLD LINES	NEW LINES IN CLEAN DOCUMENT**

| 1.There are various terms used to describe the study site and the group of animals sampled - “colony” “flying fox camp” “Adelaide grey-headed flying fox population”. Clarification of the terms used is necessary to interpret the results and understand their significance. | Thanks for making this point  We have decided to use the term camp only as it indicates that the population size can vary over the year and between years, due to births, deaths, immigration and emigration. |
| --- | --- |
| 2. From an epidemiological perspective – what is the study population? i.e. which group of flying foxes constituted the sampling frame from which the sample were drawn? The text includes “immigration of approximately 10,000 extra grey-headed flying foxes into the Adelaide camp”. Where did these bats come from? The reader needs to have a clear understanding of the group of flying foxes that could have been selected for sampling in this study, e.g. to understand the potential bias in only sampling from one location. | The study bat camp is the subset of sampled bats from our source population, which is also our target population (see below). |
| 3. what is the target population? i.e. to which group of flying-foxes are the results of this study to be applied? This is relevant to clarify when considering which previous studies are appropriate to include for comparison of results in the Discussion. And to consider the application and relevance of these results to other flying-fox populations/roosts/species etc.  The term “Adelaide colony” is used frequently in the text but it seems evident that the group of bats present at the roost site in Adelaide does not constitute a “colony” in the biological sense of the word. “Roost” or “camp” site would seem more appropriate given the evidence of long-distance travel and high level of genetic mixing of this species. I understand the two questions above may be difficult, or not possible, to answer with high confidence. But if this is the case, then this should be explicitly explained and discussed. Perhaps two or more hypotheses could be presented for potential study and target populations, and then the results and conclusions considered in light of different potential population definitions. | Thanks for the comment.  Our target (and source) population is a single group of bats physically present at a single location (at the only known camp in SA). In epidemiological term, this camp is an open population with seasonal emigration and immigration, the extent of which is not documented by any governmental or non-governmental organisation at the moment mainly because of the logistical difficulty in tracking unidentified individuals. Over the study period (2015-18) the camp size varied seasonally and year to year with increasing trend from around 4,000 in 2015 to around 20,000 in 2018.  We have changed the manuscript to use the word camp throughout. |
| 4. Regarding the demonstration of freedom of infection from ABLV:  The stated assumption of perfect accuracy of the Luminex assay seems unreasonable without strong supporting evidence. Methods are readily available for analysis of results with assumptions of imperfect sensitivity and specificity of a diagnostic test and this would seem more reasonable. | Thanks for the comments. Our main limitation does not rely on the methodology but in the access to valid estimates of Se and Sp for the Luminex. In the context of demonstration of freedom, one would be primarily concerned about the Sp of the test (i.e. possible false positives). Our data suggests (all bats tested negative) that the risk of false positive is low for this assay. In terms of Se, there is a possibility of false negatives, but we have no documented evidence of false negatives at this time, and the observed homogenous distribution of the MFI suggest otherwise (i.e. no borderline result) |
| 5. There is a statement in the Discussion that it is possible the “antigen used in the Luminex assay no longer contains a relevant epitope”. Further work to clarify this would seem necessary to interpret the results. How does the antigen in the assay compare to those found on the corresponding virus detected in Adelaide in 2012? | Thanks. We have decided to remove this sentence to improve clarity and minimise confusion. ABLV serology was not performed on the bat that died in 2012. Its unlikely there would be an epitope change. |
| 6. The freedom from infection calculations require clarification or greater explanation in the context of clarification of the definition of the “Adelaide colony” i.e. the target and study populations. Which group of bats is being considered free from infection? Are the “10,000 extra bats” included in this? | The ’open’ nature of our study population with frequent immigration/emigration (i.e., non-permanent residents) is taken into consideration in the demonstration of freedom modelling by including a ‘between-survey’ probability of exposure and/or introduction of exposed bats. This has been included and clarified in the manuscript. |
| 7. Given the statement “Freedom from Australian bat lyssavirus was deemed achieved if the estimated probability of freedom was ≥ 95%.” What does ≥ 95% mean in this context? How can this be useful? | Thanks for the comment. In other words, the probability of the camp to be free from ABLV was estimated above 95%, regardless of varying selected parameters. Here, 100% probability of freedom is realistically unachievable because of incomplete sampling coverage, therefore a widely acceptable threshold of the probability of freedom needed to be set. We used a threshold of 95% which complies with most demonstration of freedom exercises nationally and internationally. The manuscript has been reworded. |
| 8. Given the comments 4-7 above, I’m not convinced that demonstration of freedom from infection is very meaningful or useful in this context. Please provide explanation of the purpose in using the “freedom of infection” approach. | Thanks for the comment. One may think there is not much value in such a modelling exercise because the evidence of freedom is limited and applies to exposure, however, our intent was to quantify the strength of evidence for freedom from our study. Although this approach is conventionally used for infection status of population, we can assume with some confidence that showing absence of exposure is a valid evidence of absence of infection. The reverse (using infection status to demonstrate freedom of exposure) may not hold true. The manuscript has been reworded. |

**Reviewer 2 - Minor Comments** **– OLD LINES	NEW LINES IN CLEAN DOCUMENT**

| 9. The discussion on infection dynamics and previous studies is useful but could be greatly improved through clarification of the target and study populations in this study. Currently it seems rather speculative. | We hope we have clarified the discussion and the inferences from our study by removing the following sentences  This section has been reworded |
| --- | --- |
| 10. Re the statement “Uniquely, four recaptures of individuals over the sampling period provided some information on the infection dynamics of these viruses”. In what context is this unique?  Also, data from the recapture of such a small number of individuals seems more like interesting data to perhaps guide future studies rather than true information on infection dynamics. Particularly in light of the limitation of the tests used and uncertainty of the viruses to which the bats have been exposed. | Thanks – I have removed the word uniquely.  Thanks for the comment – I realise it is still difficult to infer anything specific from two time points for a given individual. Capturing more individuals on more occasions would give more useful information. This section has been reworded. |
| 11. Re the statement “there is no evidence of how long titres might last in adults”. I think some of the previous studies referred to in the Introduction and Discussion have some evidence on this. At least with regards to potential models of infection dynamics and duration of immunity. | We have adjusted the manuscript to take this into consideration. We hope this is ok |
| 12. it would be helpful to provide confidence intervals for the seroprevalences measured for ABLV in previous studies to aid interpretation of results from this study. | Thanks – these have been added to the text |
| 13. re the statement “overlap of flying fox species coupled with their range expansions, transmission and infection may occur anywhere along the distribution range”. I do not see that range expansions are required for transmission and infection to occur throughout the range of Australian flying-fox species. | Thanks  I am not trying to say that ‘range expansions are required for transmission and infection to occur throughout the flying fox range’, rather transmission and infection can occur anywhere. Have decided to remove range expansions and hope that clarifies the sentence |
| 14. re the statement “its possible that the high seropositivity to Tioman virus could reflect exposure due to either or both viruses at different times.” Given the evidence of Menangle virus and not Tioman virus presence in Australia, why test for Tioman antibodies and not Menangle antibodies? Or at least conduct a study to estimate the degree of cross reactivity of antibodies in this species. | Thanks for the comment.  By using the Tioman protein as a target we are casting a wider net to see what is possibly circulating in the bat camp. We know that this protein cross reacts with Menangle and other bat Rubulaviruses. This information could then be used for more targeted analysis |
| 15. the statements “all four species of mainland Australian pteropids” and “one of five species of flying foxes found in Australia,” appear potentially contradictory, please clarify. | Thanks – changed to flying foxes on mainland Australia only- a fifth species– the Christmas Island Flying fox is not seen on mainland Australia |
| 16. lines 325-326 I think “did not become infected” should be “did not show evidence of exposure” | Thanks  Reworded in the manuscript |
| 17. Regarding the statements on reasons for not sampling during October to December and April to May. How was the potential detrimental effect of sampling during these times determined? How is this reconciled with the stated conclusion to “include more sampling periods over an annual cycle”? | Thanks  It was difficult to obtain ethics committee approval to sample more frequently and there was concerns that sampling particularly during the birthing period would be too disruptive, leading to potential mis mothering. However, theoretically, it would be more useful to sample more frequently which is why it is suggested in the text. |
| 18. please check for minor typographical errors e.g. lines 84-85, line 405 “its” should be “it is” | Corrected |
